# Operations for the Results of Cholelithiasis

**Published:** 1910-04-02

**Authors:** 


### Surgery.

### OPERATIONS FOR THE RESULTS OF CHOLELITHIASIS.

In a previous article published on February 19, a short account was given of the operations which can be performed for an uncomplicated case of gall-stones, and it was pointed out that cholecystostomy is the best form of surgical treatment, and is preferable to cholecystectomy in the absence of certain definite indications which call for the performance of the latter operation.

### Examining the Duct.

But all cases are not so simple. It sometimes happens that after removing the stones from the gall-bladder there is some obstruction of the duct. The examination of the duct to discover whether it is patent or not should never be omitted. It should first be gently probed *via* the open gall-bladder. A large leaden probe is an admirable instrument for this purpose, because it is difficult to apply with it sufficient pressure to damage any part of the biliary apparatus. This may be done fairly easily with the ordinary probe, which is too rigid and rather too pointed to be safe. The commonest cause of obstruction is, of course, the impaction of a calculus in some part of the common duct. Sometimes the point of the probe can be felt to grit against this; but more often there is only a sense of resistance at a given point, and the nature of the obstructing body cannot be made certain. The duct should then be examined digitally from the outside—*i.e.* from its peritoneal aspect. In this way a stone can often be felt in the duct. This may be situated at any part of it, but the commonest points are: (1) just at the opening into the duodenum, and (2) where the cystic duct joins the hepatic to form the common bile-duct.

### Impacted Stones.

An impacted calculus must be removed somehow, since if a cholecystostomy is performed and the obstruction in the duct is left, a permanent biliary fistula will result, and the operation is foredoomed to failure. The ideal method of getting rid of the calculus is to push it on into the duodenum, when it will be passed and give rise to no further trouble. But this is only possible when the stone is impacted at the duodenal orifice, and not always then, since it is often situated in a sort of diverticulum which has formed round it; and again, being a single stone, its surface is rough and adherent to the mucous membrane, as opposed to the rounded or faceted stones which are found in the gall-bladder itself. If the attempts to push it on into the duodenum or alternatively to squeeze it back into the bladder fail, it will be necessary to incise the duct from its exterior and to get rid of the stone by this means (choledochotomy).

### Incising the Duct.

Choledochotomy is not in itself a difficult operation if the bile ducts can be freely exposed before the incision is made, but if the parts are matted together by adhesions the greatest trouble may be experienced. It is well to incise the duct not exactly over the situation of the impacted calculus, since the mucous membrane is often diseased at that spot, but either just above or just below it. The stone can then be extracted by milking it towards the incision. Further, the duct can be explored upwards and downwards through the incision, and this is generally rendered easy because a duct which has been obstructed is nearly always dilated, sometimes to so large a calibre that the finger can actually be introduced. In fact, if it is found when the gall-bladder is opened that the cystic duct will only just admit a large probe, it is presumptive evidence that there is no organic obstruction in it. It should be added that in making the incision the adjacent peritoneum should be protected from the possibility of infection by packing it round with gauze, since the bile contained in an obstructed duct is rarely quite aseptic. Further, the opening should only be sewn up if the gall-bladder is to be drained. Otherwise a small tube should be placed in the opening in the duct.

### Malignant Obstruction.

But the impaction of a calculus is not the only cause of duct obstruction. Malignant disease of the pancreas or of the duct itself, chronic pancreatitis, or stricture following ulceration may equally cause occlusion, and in these conditions the cause is irremovable, and the only means of affording the patient relief is by making an artificial communication between the gall-bladder itself and some part of the intestinal tract (cholecystenterostomy). In malignant disease this operation confers little benefit, and the symptoms soon recur as the disease progresses; but when done for inflammatory obstructions the results obtained are quite satisfactory. It can also be performed for persistent biliary fistula, occurring after cholecystostomy.

### Cholecystenterostomy.

The site of election for fixing the fundus of the gall-bladder is the duodenum, but if owing to the fixation of this portion of the gut the two cannot be approximated, the jejunum, or even the colon, may be chosen. Cases have been recorded in which a cholecystenterostomy has been spontaneously produced in each of these situations by the ulceration of a calculus from the gall-bladder into the alimentary tract. When this operation was first introduced it was customary to perform it by means of a Murphy's button specially made of a small size, but this has been gradually superseded by the method of simple suture, performed in exactly the same way as in gastro-enterostomy. The results thus obtained are better than those when Murphy's button was used, and there is little doubt that the method is superior.

